# A novel inflammation-based prognostic index for patients with esophageal squamous cell carcinoma: neutrophil lymphocyte ratio/albumin ratio

**DOI:** 10.18632/oncotarget.21989

**Published:** 2017-10-20

**Authors:** Qiang Zhao, Sheng Chen, Ji-Feng Feng

**Affiliations:** ^1^ Department of Thoracic Surgery, Zhejiang Cancer Hospital, Hangzhou, P.R.China; ^2^ Key Laboratory Diagnosis and Treatment Technology on Thoracic Oncology, Hangzhou, P.R. China

**Keywords:** esophageal squamous cell carcinoma, c-reactive protein, neutrophil lymphocyte ratio, cancer-specific survival, prognosis

## Abstract

**Background:**

We initially proposed a novel inflammation-based prognostic index, named neutrophil lymphocyte ratio/albumin ratio (NLR/Alb), for predicting the postoperative survival in esophageal squamous cell carcinoma (ESCC).

**Materials and methods:**

A retrospective study of 329 cases with resectable ESCC was included. The optimal cut-off values were evaluated by X-tile program. The 5-year cancer-specific survival (CSS) was calculated by Kaplan–Meier method. Cox regression analyses were performed to evaluate the prognostic factors.

**Results:**

The optimal cut-off value was 0.1 for NLR/Alb according to the X-tile program. There was a significantly better 5-year CSS in patients with NLR/Alb ≤ 0.1 than patients with NLR/Alb > 0.1 (39.1% vs. 11.0%, *P* < 0.001). According to multivariate analyses, NLR/Alb (*P =* 0.001) was an independent prognostic factor.

**Conclusions:**

The NLR/Alb is a novel and usefull predictive factor in patients with ESCC.

## INTRODUCTION

Esophageal cancer (EC) is one of the most fatal types of cancer worldwide [1, 2]. Esophageal squamous cell carcinoma (ESCC) is the major pathological type in China [3, 4]. Although the treatment has been improved in recent years, the prognosis for EC remains poor. Therefore, assessing the prognostic factors in patients with EC will become more important. To date, several biomarkers have been evaluated to predict the prognosis for EC, but the prognostic values remain controversial [5].

Inflammation plays an important role in cancer progression and prognosis [6, 7]. Therefore, a series of inflammatory biomarkers, such as c-reactive protein (CRP), neutrophil lymphocyte ratio (NLR) and Glasgow prognostic score (GPS) have been evaluated to predict the prognosis in several cancers [8–12]. Recently, the CRP/Albumin (CRP/Alb) ratio was reported to correlate with prognosis in various cancers, including EC [13–15]. However, to our knowledge, no study so far has assessed the clinical significance of the NLR/Albumin (NLR/Alb) ratio in other cancers as well as EC. Therefore, the aim of this study was to investigate the prognostic value of NLR/Alb ratio in patients with resectable ESCC.

## RESULTS

### Patient characteristics

**Figure 1:**
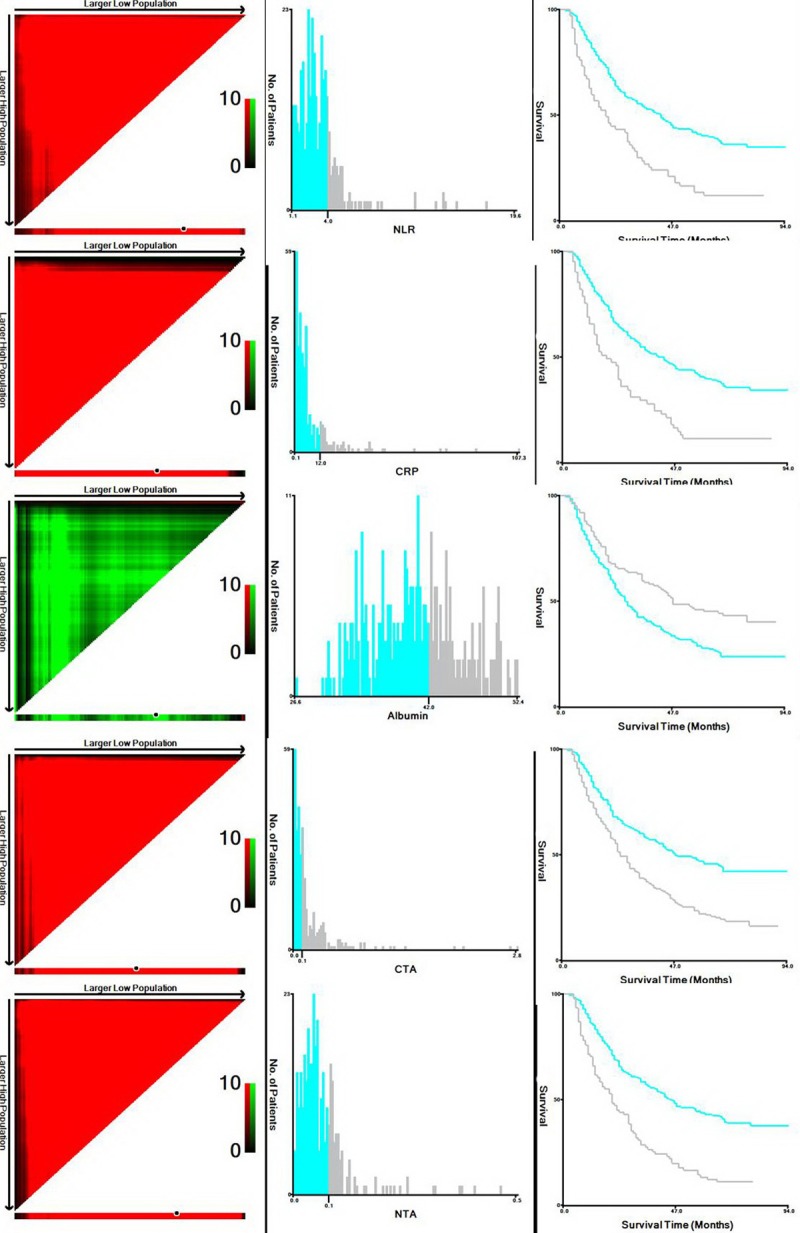
X-tile analyses X-tile plots of the training sets are shown in the left panels, with plots of matched validation sets shown in the smaller inset. The optimal cut-off point highlighted by the black circle in the left panels is shown on a histogram of the entire cohort (middle panels), and a Kaplan–Meier plot (right panels). According to the X-tile program, the optimal cut-off values for NLR, CRP, albumin, CRP/Alb and NLR/Alb were 4.0, 12.0 mg/l. 42.0 g/l, 0.1 and 0.1, respectively. (NLR = neutrophil lymphocyte ratio; CRP = c-reactive protein; CTA = CRP to albumin; NTA = NLR to albumin)

Of the total number of cases, 42 (12.8%) were women and 287 (87.2%) were men. According to the X-tile program, the optimal cut-off values for NLR, CRP, albumin, CRP/Alb and NLR/Alb were 4.0, 12.0 mg/l. 42.0 g/l, 0.1 and 0.1, respectively (Figure 1). According to the cut-off value, then patients were divided into two groups: NLR/Alb ≤ 0.1 and NLR/Alb > 0.1. The relationships between NLR/Alb and clinical characteristics were shown in Table 1. Our study revealed that NLR/Alb ratio was associated with tumor length (*P* < 0.001), TNM stage (*P* < 0.001), NLR (*P* = 0.021), CRP (*P* = 0.002), Alb (*P* < 0.001), CRP/Alb (*P* = 0.002) and GPS (*P* < 0.001), respectively.

**Table 1:** The relationship between NLR/Alb and clinical characteristics for ESCC

	Total (*N* = 329) (*n*, %)	NLR/Alb ≤ 0.1 (*N* = 238) (*n*, %)	NLR/Alb > 0.1(*N* = 91) (*n*, %)	*P*-value
Sex Male Female	287 (87.2) 42 (12.8)	207 (87.0) 31 (13.0)	80 (87.9) 11 (12.1)	0.820
Age (years) ≤ 60 > 60	190 (57.8) 139 (42.2)	137 (57.6) 101 (42.4)	53 (58.2) 38 (41.8)	0.911
Tumor length (cm) ≤ 3.0 > 3.0	92 (28.0) 237 (72.0)	82 (34.5) 156 (65.5)	10 (11.0) 81 (89.0)	< 0.001
Tumor location Upper Middle Lower	19 (5.8) 154 (46.8) 156 (47.4)	14 (5.9) 117 (49.2) 107 (44.9)	5 (5.5) 37 (40.7) 49 (53.8)	0.345
Vessel invasion Positive Negative	51 (15.5) 278 (84.5)	38 (16.0) 200 (84.0)	13 (14.3) 78 (85.7)	0.706
Perineural invasion Positive Negative	64 (19.5) 265 (80.5)	47 (19.7) 191 (80.3)	17 (18.7) 74 (81.3)	0.827
Differentiation Well Moderate Poor	46 (14.0) 220 (66.9) 63 (19.1)	32 (13.4) 164 (68.9) 42 (17.7)	14 (15.4) 56 (61.5) 21 (23.1)	0.422
TNM stage I II III	85 (25.8) 109 (33.1) 135 (41.1)	77 (32.4) 83 (34.9) 78 (32.7)	8 (8.8) 26 (28.6) 57 (62.6)	< 0.001
Adjuvant therapy Yes No	94 (28.6) 235 (71.4)	63 (26.5) 175 (73.5)	31 (34.1) 60 (65.9)	0.173
NLR (mean ± SD) ≤ 4.0 > 4.0	3.36 ± 2.27 220 (66.9) 109 (33.1)	2.56 ± 1.25 201 (84.5.) 37 (15.5)	5.45 ± 2.92 19 (20.9.) 72 (80.1)	< 0.001^*^ < 0.001
CRP (mg/l, mean ± SD) ≤ 12.0 > 12.0	7.42 ± 11.8 264 (80.2) 67 (19.8)	6.20 ± 10.60 201 (84.5) 37 (15.5)	10.6 ± 14.1 63 (69.2) 28 (30.8)	0.007^*^ 0.002
Albumin (g/l, mean ± SD) ≤ 42.0 > 42.0	40.51 ± 5.30 205 (62.3) 124 (37.7)	41.77 ± 4.85 129 (54.2) 109 (45.8)	37.20 ± 5.04 76 (83.5) 15 (16.5)	< 0.001^*^ < 0.001
CRP/Alb (mean ± SD) ≤ 0.1 > 0.1	0.19 ± 0.33 175 (53.2) 154 (46.8)	0.15 ± 0.27 139 (58.4) 99 (41.6)	0.30 ± 0.43 36 (39.6) 55 (60.4)	< 0.001^*^ 0.002
GPS 0 1 2	194 (59.0) 95 (28.9) 40 (12.1)	164 (68.9) 65 (27.3) 9 (3.8)	30 (33.0) 30 (33.0) 31 (34.0)	< 0.001

ESCC = esophageal squamous cell carcinoma; NLR = neutrophil lymphocyte ratio; Alb = albumin; CRP = c-reactive protein; GPS= Glasgow prognostic score Statistical methods: The chi-squared test. “^*^” by *t*-test.

### Pearson correlation

**Figure 2:**
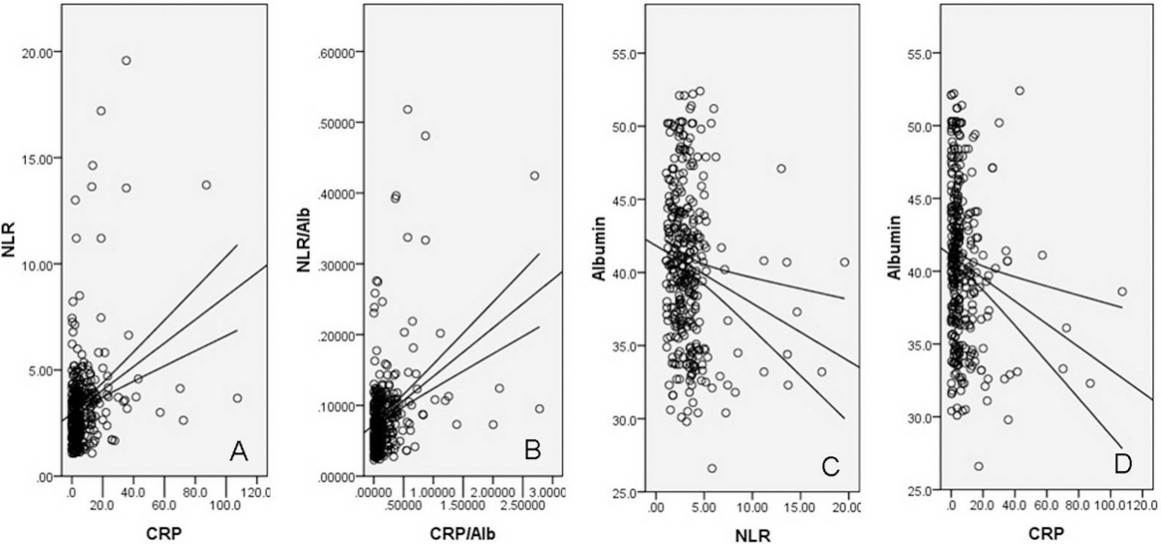
Pearson correlation analysis Positive correlations between NLR and CRP (r = 0.288, *P* < 0.001, (**A**), between NLR/Alb and CRP/Alb (r = 0.351, *P* < 0.001, (**B**); negative correlations between NLR and Alb (r = -0.169, *P* = 0.002, (**C**), between CRP and Alb (r = –0.175, *P* = 0.001, (**D**).

Pearson correlation analyses revealed that there were significant positive correlations between NLR and CRP (r = 0.288, *P* < 0.001, Figure 2A), NLR/Alb and CRP/Alb (r = 0.351, *P* < 0.001, Figure 2B), but negative correlations between NLR and Alb (r = –0.169, *P* = 0.002, Figure 2C), CRP and Alb (r = –0.175, *P* = 0.001, Figure 2D).

### Kaplan–Meier analyses

**Figure 3:**
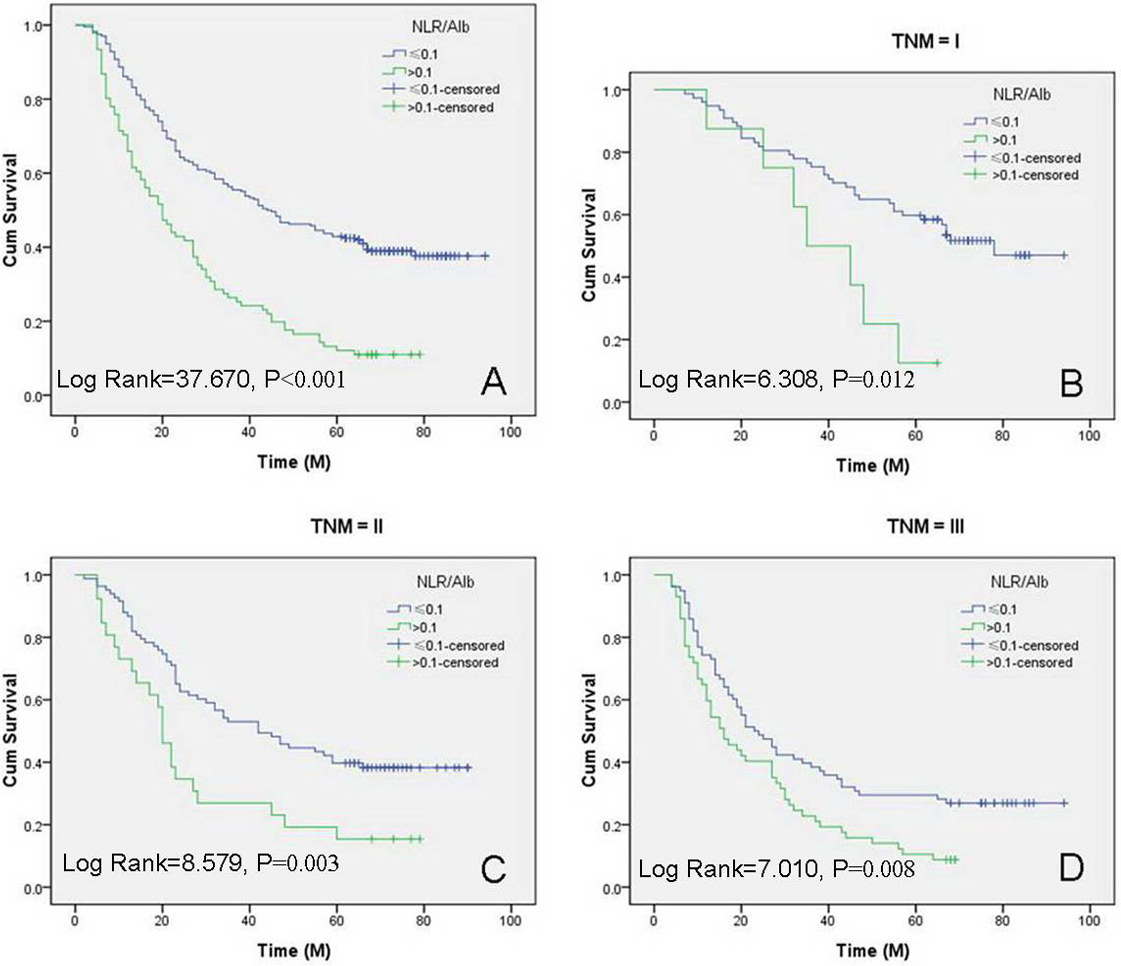
Kaplan–Meier CSS curves stratified by NLR/Alb The range duration of follow-up were 3 to 94 months, with the median of 34 months. A significantly better 5-year CSS in patients with NLR/Alb ≤ 0.1 than patients with NLR/Alb > 0.1 (39.1% vs. 11.0%, *P* < 0.001; median CSS: 44 months vs. 20 months; (**A**). Patients with NLR/Alb ratio ≤ 0.1 had a significantly better 5-year CSS than patients with NLR/Alb ratio > 0.1 in TNM I (51.9% vs. 12.5%, *P* = 0.012; median CSS: 78 months vs. 35 months; (**B**), TNM II (38.6% vs. 15.4%, *P* = 0.003; median CSS: 42 months vs. 20 months; (**C**) and TNM III (26.9% vs. 8.8%, *P* = 0.008; median CSS: 23 months vs. 16 months; (**D**).

Kaplan–Meier analyses demonstrated that there was a significantly better 5-year CSS in patients with NLR/Alb ≤ 0.1 than patients with NLR/Alb > 0.1 (39.1% vs. 11.0%, *P* < 0.001) (Figure 3A). Then, we further stratified patients into different groups based on TNM stage. Our results demonstrated that NLR/Alb ratio was also significantly correlated with CSS based on TNM stage (Figure 3B–3D).

### Univariate and multivariate analyses

In univariate analyses, tumor length (*P* = 0.030), vessel invasion (*P* = 0.010), perineural invasion (*P* = 0.034), TNM stage (*P* < 0.001), NLR (*P* < 0.001), CRP (*P* < 0.001), Alb(*P* = 0.001), GPS (*P* < 0.001), CRP/Alb (*P* < 0.001) and NLR/Alb (*P* < 0.001) were also significant predictors of CSS. Multivariate analyses revealed that NLR/Alb (*P* = 0.001) was an independent prognostic factor (Table 2). In addition, TNM stage (*P* < 0.001) and CRP/Alb (*P* < 0.001) were also significant independent predictors for CSS (Table 2).

**Table 2:** Univariate and multivariate analyses for cancer-specific survival

	Univariate analyses	*P*-value	Multivariate analyses	*P*-value
	HR (95% CI)		HR (95% CI)	
Sex (male vs female)	1.055 (0.710–1.567)	0.791		
Age (years, > 60 vs ≤ 60)	1.023 (0.785–1.332)	0.868		
Tumor length (cm, > 3.0 vs ≤ 3.0)	1.391 (1.032–1.876)	0.030		
Tumor location Upper Middle Lower	1.000 (Reference) 1.311 (0.705–2.440) 1.302 (0.700–2.422)	0.687 0.392 0.404		
Vessel invasion (yes vs no)	1.562 (1.112–2.192)	0.010		
Perineural invasion (yes vs no)	1.406 (1.026–1.926)	0.034		
Differentiation Well Moderate Poor	1.000 (Reference) 1.183 (0.790–1.770) 1.492 (0.931–2.392)	0.216 0.415 0.097		
TNM stage I II III	1.000 (Reference) 1.696 (1.166–2.467) 2.654 (1.866–3.774)	< 0.001 0.006 < 0.001	1.000 1.481 (1.014–2.163) 2.129 (1.474–3.074)	< 0.001 0.042 < 0.001
Adjuvant therapy (yes vs no)	1.116 (0.836–1.488)	0.457		
NLR (> 4.0 vs ≤ 4.0)	2.130 (1.579–2.872)	< 0.001		
CRP (mg/l, > 12.0 vs ≤ 12.0)	2.037 (1.502–2.761)	< 0.001		
Albumin (g/l, > 42.0 vs ≤ 42.0)	0.619 (0.467–0.820)	0.001		
CRP/Alb (> 0.1 vs ≤ 0.1)	1.919 (1.473–2.498)	< 0.001	1.640 (1.248–2.154)	< 0.001
GPS 0 1 2	1.000 (Reference) 1.960 (1.465–2.623) 2.463 (1.676–3.619)	< 0.001 < 0.001 < 0.001		
NLR/Alb (> 0.1 vs ≤ 0.1)	2.293 (1.741–3.020)	< 0.001	1.672 (1.245–2.245)	0.001

ESCC = esophageal squamous cell carcinoma; NLR = neutrophil lymphocyte ratio; Alb = albumin; CRP = c-reactive protein; GPS = Glasgow prognostic score; HR = hazard ratio; CI = confidence interval Statistical methods: Univariate and multivariate analyses.

### ROC analysis

**Figure 4:**
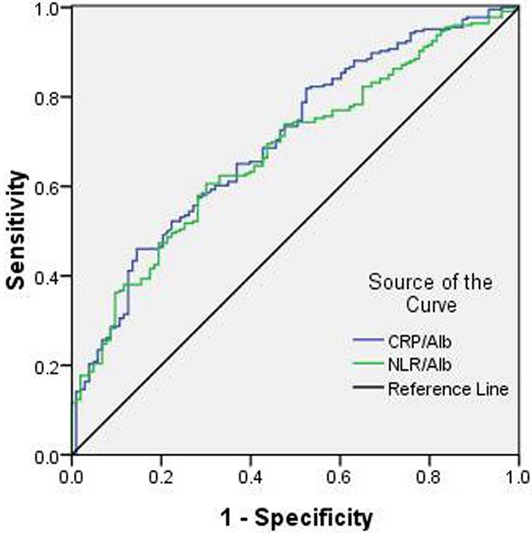
ROC curves for CSS prediction A ROC curve plots the sensitivity on the y-axis against one minus the specificity on the x-axis. A diagonal line at 45 degrees, known as the line of chance, would result from a test which allocated subjects randomly. Each point on the ROC cueve corresponds to a value by Youden Index (sensitivity+specificity-1). The areas under the curve (AUC) was 0.702 (95% CI: 0.635-0.763, *P* < 0.001) for CRP/Alb and 0.678 (95% CI: 0.574-0.716, *P* < 0.001) for NLR/Alb, respectively.

The areas under the curve (AUC) was 0.702 (95% CI: 0.635–0.763, *P* < 0.001) for CRP/Alb and 0.678 (95% CI: 0.574–0.716, *P* < 0.001) for NLR/Alb, respectively. The discrimination ability of the NLR/Alb was similar to CRP/Alb, indicating that NLR/Alb predicts survival in ESCC similar to CRP/Alb (Figure 4).

## DISCUSSION

As we know, both NLR and Alb are influenced by various conditions, and the ratio of NLR and Alb could therefore minimise the potential basis. To our knowledge, this is the first study to evaluate the prognostic role of NLR/Alb ratio in predicting prognosis for patients with resectable ESCC. In the present study, therefore, a novel inflammation-based prognostic index was conducted based on NLR and Alb and was shown to be an independent predictor for patients with resectable ESCC.

There is strong linkage between inflammation and cancer. As we know, serum CRP is a very sensitive indicator of systemic inflammatory response, and NLR is other index of systemic inflammation [16]. Previous studies have shown that serum CRP and NLR are predictors of prognosis in several cancers, including EC [8–10]. Recently, we conducted two meta-analyses [17, 18] revealed that CRP and NLR were significantly associated with prognosis in patients with EC. In the current study, however, CRP or NLR were not independent prognostic factors in multivariate analyses.

Recently, the CRP/Alb ratio was reported to correlate with prognosis in patients with various cancers, including EC [13–15]. Wei [14] and Xu [15] revealed that CRP/Alb ratio is a novel and usefull predictive factor in patients with ESCC. In the current study, CRP/Alb ratio was still an independent prognostic factor (*P* < 0.001). In the current study, therefore, we firstly investigated the prognostic significance of NLR/Alb ratio in ESCC patients. Recently, Camp [19] initial developed a graphical method, named X-tile, to illustrate a method of dividing a single cohort into training and validation subsets. They concluded that the X-tile plot can present a new tool for the assessment of biological relationships and discover cut-points based on marker expression. After that, more and more researchers have revealed that the X-tile is a robust graphical tool, to determine the optimal cut-off values [20–22]. In the current study, the optimal cut-off value for NLR/Alb was calculated by the X-tile program, which was 0.1. Kaplan–Meier analyses demonstrated that there was a significantly better 5-year CSS in patients with NLR/Alb ≤ 0.1 than patients with NLR/Alb > 0.1 (39.1% vs. 11.0%, *P* < 0.001). Multivariate analyses revealed that NLR/Alb (*P* = 0.001) was an independent prognostic factor.

In the current study, the GPS, a well-known inflammatory parameter, was not an independent prognostic factor. NLR/Alb (*P* = 0.001) and CRP/Alb (*P* < 0.001) were significant independent predictors for CSS. The areas under the curve (AUC) was 0.702 (95% CI: 0.635–0.763, *P* < 0.001) for CRP/Alb and 0.678 (95% CI: 0.574–0.716, *P* < 0.001) for NLR/Alb, respectively. The discrimination ability of the NLR/Alb was similar to CRP/Alb, indicating that NLR/Alb predicts survival in ESCC similar to CRP/Alb.

There is no consensus for the standard treatment for EC. However, esophagectomy with lymph node dis remains the standard treatment for patients with early stage. Recent trials, including CROSS, MAGIC, ACCORD and OEO2, have established neoadjuvant therapy as standard of care for locally advanced esophageal cancer [23–27]. In addition, studies have demonstrated that postoperative adjuvant chemoradiotherapy significantly improves the long-term survival of patients with EC compared with surgery alone [28, 29]. However, the current study was a retrospective study between 2005 and 2008, the preoperative adjuvant radiotherapy and/or chemotherapy was controversial at the time. On the other hand, patients who received preoperative neoadjuvant treatment were excluded because neoadjuvant therapy will have an important impact on the inflammation. However, no significant difference was found regarding 5-year CSS in adjuvant therapy in our study. Two possible reasons were as follows: Firstly, the postoperative adjuvant chemoradiotherapy was not mandatory in our study. Secondly, most of patients with postoperative adjuvant therapy were locally advanced disease.

Several limitations should be acknowledged in the current study. Firstly, the current study was a retrospective study with a small sample. Secondly, in our study, we excluded patients who had received neoadjuvant treatment, which may have influenced the result. The results of the study should therefore be regarded with caution. Therefore, larger prospective studies will need to be performed to confirm the prognostic value for NLR/Alb ratio. 

In summary, based on the findings of the current study, the NLR/Alb ratio is a usefull and independent predictive factor in patients with ESCC. The NLR/Alb is easy to measure routinely because of its low cost and convenience, which can be used in clinical practice as a sensitive cancer biomarker. We also conclude that 0.1 may be the optimal cut-off point for NLR/Alb ratio in predicting CSS in patients with ESCC.

## MATERIALS AND METHODS

### Patients

Between January 2005 and December 2008, a total of 329 consecutive cases with resectable ESCC were included in the current retrospective study. The eligibility criteria were included: (1) ESCC was confirmed by histopathological examination; (2) curative surgery with margins histologically free of disease; and (3) preoperative serum NLR, CRP and Alb were obtained before surgery within one week. Exclusion criteria were as follows: patients who received preoperative neoadjuvant treatment, patients who had distant metastasis and those who had any form of acute infection or chronic inflammatory disease.

### Treatment

All patients underwent curative esophagectomy. The standard surgical approach included the Ivor Lewis and the McKeown procedure with two-field or three-field lymphadenectomy [23, 24]. Patients who had received neoadjuvant therapy were excluded in the current study. As the role of postoperative adjuvant treatment was controversial during that period, adjuvant therapy was not mandatory. The most frequent adjuvant chemotherapy included cisplatin and 5-fluorouracil. The median postoperative radiation dose was 50 Gy.

### Data collection

Blood samples were obtained before surgery to measure CRP, neutrophil count, lymphocyte count and albumin levels. Data on preoperative laboratory examination were extracted in our medical records. The serum levels of NLR, CRP, Alb, NLR/Alb and CRP/Alb were taken within one week prior to surgery.

### Follow-up

The patients in the current study were staged according to the 7th edition of the American Joint Committee on Cancer Cancer Staging [30]. The study was approved by the Ethics Committees of Zhejiang Cancer Hospital. In the current study, a cancer-specific survival (CSS) analysis was ascertained. The last follow-up was 30 June 2013.

### Statistical analysis

The optimal cut-off values for NLR, CRP, albumin, CRP/Alb and NLR/Alb were calculated by a X-tile program [19]. Pearson correlation analyses were performed to calculate the correlation among CRP, NLR, Alb, CRP/Alb and NLR/Alb. A *t*-test and chi-squared test were used to determine the significance of differences for patients grouped by NLR/Alb. Kaplan–Meier methods were used to analyse CSS. Univariate and multivariate analyses were performed to analyse the prognostic factors. A receiver operating characteristic (ROC) curve for CSS prediction was plotted. The area under curve (AUC) was used as an estimation of diagnostic accuracy. Statistical analyses were conducted with SPSS 17.0 (SPSS Inc., Chicago, IL, USA).
